# CDK11 Promotes Cytokine-Induced Apoptosis in Pancreatic Beta Cells Independently of Glucose Concentration and Is Regulated by Inflammation in the NOD Mouse Model

**DOI:** 10.3389/fimmu.2021.634797

**Published:** 2021-02-10

**Authors:** Ester Sala, Celia Vived, Júlia Luna, Noemí Alejandra Saavedra-Ávila, Upasana Sengupta, A. Raúl Castaño, Sabrina Villar-Pazos, Laura Haba, Joan Verdaguer, Ana B. Ropero, Thomas Stratmann, Javier Pizarro, Manuel Vázquez-Carrera, Angel Nadal, Jill M. Lahti, Conchi Mora

**Affiliations:** ^1^ Immunology Unit, Department of Experimental Medicine, Faculty of Medicine, University of Lleida, Lleida, Spain; ^2^ Institut de Recerca Biomèdica Lleida (IRB-LLeida), Lleida, Spain; ^3^ Departament of Cell Biology, Physiology and Immunology, Autonomous University of Barcelona, Barcelona, Spain; ^4^ Instituto de Investigación, Desarrollo e Innovación en Biotecnología Sanitaria de Elche, IDiBE, Universidad Miguel Hernandez, Elche, Spain; ^5^ Department of Tumor Cell Biology, St. Jude Children’s Research Hospital, Memphis, TN, United States; ^6^ Experimental Diabetes Laboratory, Institute for Biomedical Research August Pi i Sunyer (IDIBAPS), Barcelona, Spain; ^7^ Instituto de Bioingeniería, Universidad Miguel Hernández, Elche, Spain; ^8^ Department of Cell Biology, Physiology and Immunology, Faculty of Biology, University of Barcelona, Barcelona, Spain; ^9^ Department of Pharmacology, Toxicology and Therapeutic Chemistry, Faculty of Pharmacy and Food Sciences and Institute of Biomedicine (IBUB), University of Barcelona, Barcelona, Spain; ^10^ Spanish Biomedical Research Center in Diabetes and Associated Metabolic Diseases (CIBERDEM)—Instituto de Salud Carlos III, Madrid, Spain; ^11^ Pediatric Research Institute, Hospital Sant Joan de Déu, Esplugues de Llobregat, Spain; ^12^ Diabetes and Associated Metabolic Disorders CIBERDEM, Universidad Miguel Hernández de Elche, Elche, Spain

**Keywords:** apoptosis, beta cell, CDK11, cyclin D3, inflammation, type 1 diabetes, glucose, insulin

## Abstract

**Background:**

Pancreatic islets are exposed to strong pro-apoptotic stimuli: inflammation and hyperglycemia, during the progression of the autoimmune diabetes (T1D). We found that the *Cdk11(Cyclin Dependent Kinase 11) is* downregulated by inflammation in the T1D prone NOD (non-obese diabetic) mouse model. The aim of this study is to determine the role of CDK11 in the pathogenesis of T1D and to assess the hierarchical relationship between CDK11 and Cyclin D3 in beta cell viability, since Cyclin D3, a natural ligand for CDK11, promotes beta cell viability and fitness in front of glucose.

**Methods:**

We studied T1D pathogenesis in NOD mice hemideficient for CDK11 (N-HTZ), and, in N-HTZ deficient for Cyclin D3 (K11HTZ-D3KO), in comparison to their respective controls (N-WT and K11WT-D3KO). Moreover, we exposed pancreatic islets to either pro-inflammatory cytokines in the presence of increasing glucose concentrations, or Thapsigargin, an Endoplasmic Reticulum (ER)-stress inducing agent, and assessed apoptotic events. The expression of key ER-stress markers (*Chop*, *Atf4* and *Bip*) was also determined.

**Results:**

N-HTZ mice were significantly protected against T1D, and NS-HTZ pancreatic islets exhibited an impaired sensitivity to cytokine-induced apoptosis, regardless of glucose concentration. However, thapsigargin-induced apoptosis was not altered. Furthermore, CDK11 hemideficiency did not attenuate the exacerbation of T1D caused by Cyclin D3 deficiency.

**Conclusions:**

This study is the first to report that CDK11 is repressed in T1D as a protection mechanism against inflammation-induced apoptosis and suggests that CDK11 lies upstream Cyclin D3 signaling. We unveil the CDK11/Cyclin D3 tandem as a new potential intervention target in T1D.

## Introduction

In T1D, the proinflammatory milieu has been reported to impair the functioning of beta cells and induce apoptosis (1–4). One of the best animal models of T1D is the NOD (Non-Obese Diabetic) mouse model (5). A series of reports have focused on studying the effects of the main proinflammatory cytokines alone or in combination on the proteome/transcriptome of insulinoma/islet cells *in vitro* (6–9). Those reports included a discrete combination of cytokines that partially resembles the complex inflammatory niche hosting the pancreatic islet; and most studies used insulinoma tumor cell lines. Therefore, we explored the *in vivo* effects of inflammation on the transcriptome of NOD endocrine cells by applying cDNA microarray technology (10) and found that the mRNA expression of *Cdk11* (cyclin-dependent kinase 11) was downregulated in the pancreatic endocrine cells (PEC) from NOD female mice upon inflammation. CDK11 is a ubiquitous PITSLRE cyclin-dependent kinase with two gene products: p58 and p130 in mice, and p58 and p110 in humans (11). The p58 gene product originates from a differential ribosome entry through an IRES (Internal Ribosomal Entry Sequence) in the mRNA of *Cdk11* during its translation in mitosis (12).

CDK11^p110^ binds to cyclin L, which is the main CDK11^p110^ regulatory subunit, and several pre-mRNA splicing factors to regulate pre-mRNA transcription and processing in proliferating cells (13–17). CDK11^p110^ is expressed during all cell cycle phases (11), while CDK11^p58^ is only expressed during mitosis (18). CDK11^p58^ is required during mitosis for the maintenance of the sister chromatid cohesion and participates in cytokinesis and mitosis termination (18, 19). The activity of CDK11^p58^ depends on its interaction with Cyclin D3 to promote its kinase activity, migrate into the nucleus and regulate the cell cycle progression through the G2/M phase (20, 21). The CDK11-deficiency in mice is embryonically lethal, while CDK11-hemideficient mice are viable (22).

Furthermore, CDK11^p58^ is associated with the downregulation of the expression of Bcl-2 during apoptosis (23), and both CDK11^p58^ and CDK^p110^ are activated by Caspase-3 mediated cleavage into CDK11^p46^, in response to pro-apoptotic stimuli, which amplifies the apoptotic process through phosphorylation of regulating proteins such as the eukaryotic Initiation Factor 3f (eIF3f) (24–26). Moreover, CDK11^p110^ cleavage by Caspase 3 releases CDK11^p60^, a regulatory moiety that mediates cytochrome c release from mitochondria and apoptosis (27). Since islet beta cells undergo cytokine-induced apoptosis in T1D (28, 29), CDK11 cleavage by Caspase-3 is expected to exacerbate apoptosis in beta cells. Therefore, since beta cell replication has been reported to occur in response to the autoimmune attack (30), mitotic beta cells would express CDK11^p58^, which, in turn, would amplify apoptosis.

Moreover, beta cells synthesize insulin upon demand; this submits them to Endoplasmic Reticulum (ER)-stress and the Unfolded Protein Response (UPR). In T1D, insidious insulitis and hyperglycemia induce ER-stress and UPR activation, leading to beta cell apoptosis through the upregulation of CHOP (C/EBP homologous protein), a key transcription factor responsible for ER-stress induced-apoptosis through Bcl-2 inhibition (31, 32). In T1D, both, inflammation and ER-stress crosstalk and feed-back each other positively, resulting in exacerbation of beta cell death (33).

In this study, we aim to explore whether there is a causal relationship between the downregulation of *Cdk11* in beta cells and the onset of T1D. Moreover, we aim to determine whether the simultaneous downregulation of both, *Cdk11* and *Cyclin D3* at the onset of T1D has a pathophysiological meaning. To this end, we developed CDK11-hemideficient NOD mice and assessed their phenotype in terms of beta cell viability, autoimmune repertoire, and autoimmune diabetes. Furthermore, we developed NOD mice that were hemideficient in CDK11 and deficient in Cyclin D3 and assessed their phenotype in terms of T1D onset.

## Materials and Methods

### Mice

Mice were housed under specific pathogen-free (SPF) conditions. All animal experimentation procedures performed in this study were overseen and approved by the Institutional Ethical Committee for Animal Experimentation of the University of Lleida (CEEA) in accordance with European and U.S.A. regulations on Animal Experimentation. All results involving animal experiments were obtained with female mice. Mice heterozygous for the CDK11-deficiency (HTZ) with the mixed 129SvxC57BL/6 genetic background (22) were backcrossed to the NOD background for 15 generations to identify the *Idd* susceptibility loci (34). The null mutation of *Cdk11* was detected by PCR (22) using the CPrev: CAAGAGAAGCCTGAGCAAATAG and the mp70: GAGATACTCTTTACATGCCAACC primers. Cyclin D3 deficient mice in the NOD background (D3KO) were genotyped by PCR as previously reported (10).

### Quantitative Real-Time PCR (qRT-PCR)

See **Supplementary Methods**.

### Assessment of Pancreatic Infiltration

Immunohistochemical analysis of pancreatic infiltration was performed on paraffin sections by hematoxylin & eosin counterstaining (Sigma-Aldrich, St. Louis, MO, USA) for the infiltration studies (Leica, Wetzler, Germany). To score the islet infiltration, the following values were assigned: 0 for non-infiltrated islets, 1 for peri-insulitic lesions, 2 for intra-insular insulitis with less than 50% of the islet area infiltrated, and 3 for insulitis with more than 50% of the islet area infiltrated. A minimum of eight sections per mouse at four different levels (>150 islets per genotype) were examined. For each individual mouse, the following formula was applied to calculate the infiltration score, and then, the mean ± SEM of each genotype was plotted:

$$\frac{\left(X\;islets\;of\;score\;0\times 0)+(Y\;islets\;of\;score\;1\times 1)+(Z\;islets\;of\;score\;2\times 2)+(W\;islets\;of\;score\;3\times 3\right)}{\left(X+Y+Z+W\right)}$$

### Diabetes Assessment, Adoptive Transfer Experiments, and Pancreatic Islet Isolation

All these procedures were performed as previously described (34).

### Magnetic Cell Separation of Pancreatic Endocrine Cells (PECs: CD45^-^ Fraction)

Islets were isolated and disaggregated by trypsin digestion, and the resulting cell suspension was incubated with an anti-mouse CD45-phycoerythrin (PE) antibody (Becton Dickinson (BD), CA, USA), subsequently washed and incubated with the anti-PE antibody coupled to magnetic beads, and submitted to AUTOMACS magnetic negative selection according to the manufacturer’s instructions (AUTOMACS; Miltenyi Biotec, Bergisch Gladbach, Germany).

### Western Blot Assays

See **Supplementary Methods**.

### Islet Cell Staining

The isolated islets were trypsinized, fixed and permeabilized prior to the cellular staining with the anti-mouse Glut-2 (beta-cell marker; R&D Minneapolis, MN, USA), anti-mouse Ki67 (proliferation marker; Dako, Glostrup, Denmark), phycoerythrin-conjugated CD45 (hematopoietic marker; BD), and 7AAD (cell death marker; BD) antibodies. The data were acquired using a FACSCanto II flow cytometer (BD).

### TUNEL Assays

Determination of late (TUNEL) apoptotic events was performed using the In Situ Cell Death Detection Kit, Fluorescein (ref, 11684795910, Roche-Sigma). Insulin was detected with anti-insulin antibody (A0564, Dako), and nuclear staining was performed with Hoechst 33342 (B2261, Sigma).

### Isolation of Islet-Infiltrating Lymphocytes (IILS)

The islet-infiltrating lymphocytes (IILs) were isolated by trypsinization of the previously isolated islets using 500 µl of trypsin/EDTA (Lonza, Verviers, Belgium) for 5 min at 37°C to obtain single cell suspensions.

### Assessment of Immune Cell Subsets by Flow Cytometry

See **Supplementary Methods**.

### DNA Fragmentation Assays

Handpicked islets were cultured in supplemented DMEM in the presence or absence of a cytokine cocktail containing IFN-γ 100U/ml, TNF-α 1000 U/ml, IL1β 100U/ml, afterwards islets were harvested and lysed by hypotonic shock, stained with Propidium Iodide (Sigma) and acquired by flow cytometry to measure nuclei fragmentation (% ΔSubG1) as previously reported (35). Results were calculated as percentage on increment in SubG1 induction by cytokines as follows:

$$\frac{\left(\%SubG1 events upon exposure to cytokines)-(\%SubG1 events in basal condition\right)}{\left(\%SubG1 in basal condition\right)}\times 100$$

### ER-Stress Induction

Handpicked islets from 5–7-week-old female mice donors were cultured in supplemented DMEM containing 11mM glucose, either in the presence or absence of 1μM Thapsigargin (Sigma) for the indicated time (either 24 h for ER-stress marker induction or 96 h for subG1 measurements).

### Statistical Analysis

Mann-Whitney *U*-tests were performed to evaluate the differences between pairs of groups. The log-rank test was applied to assess the difference between the diabetes cumulative incidence curves. The one-way ANOVA test was used to assess the differences in TUNEL studies. The two-way ANOVA test was used to analyze the differences in infiltration score studies, and SubG1 studies respectively. All analyses were performed using the GraphPad Prism (v5.0a) statistical package. The threshold for statistical significance was set at 0.05.

## Results

### Cdk11 Is Downregulated in β Cells by Autoimmune Insulitis in a Dose-Dependent Manner Without Impairing β Cell Proliferation

**Figure 1:**
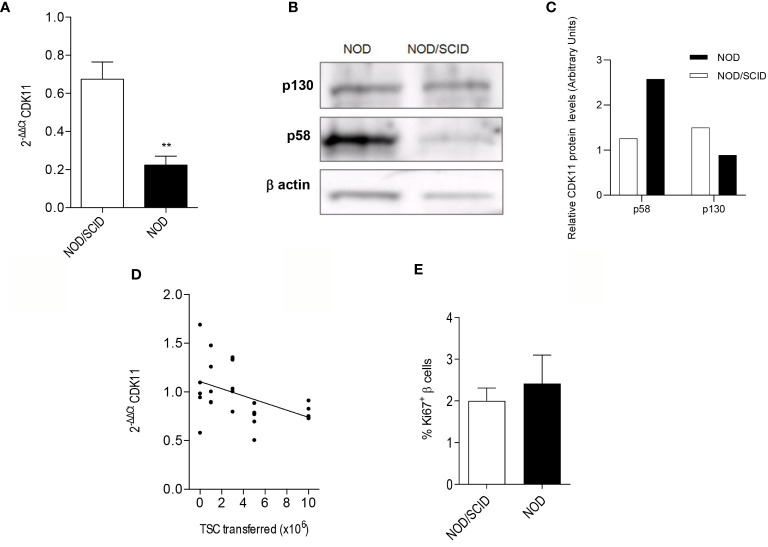
Cdk11 is downregulated by the islet infiltration at the mRNA level, without affecting the proliferative activity of β cells in NOD mice. CDK11p58 protein expression is enhanced, while CDK11p130 is downregulated, compared to NOD/SCID in NOD PECs **(A)**. Pancreatic islet cells from either 11-week-old NOD female mice (n = 6) or 11-week-old NOD/SCID female mice (n = 10) were extracted, and the CD45^-^ cell subset (PECs) was selected by magnetic sorting to perform a qRT-PCR analysis to assess the expression of *cdk11*. **(B**, **C)** Pancreatic islet cells from either 11-week-old NOD female mice (n = 16 mice; 1212 islets) or 11-week-old NOD/SCID female mice (n = 15 mice; 1586 islets) were extracted, and the CD45^-^ cell subset (PECs) was selected by magnetic sorting to perform western blot analysis to detect both CDK11p58 and CDK11p130 proteins. **(C)** CDK11p58 and CDK11p130 expression values were respectively normalized to β-actin levels in PECs from either NOD (black columns) or NOD/SCID (white columns). **(D)** Assessment of the expression levels of *cdk11* in PECs from NOD/SCID mice that were adoptively transferred with different amounts (×10^6^) of total splenocytes (TSC (i.e., total spleen cells that were previously depleted of red blood cells) from 8-week-old NOD female donors; the numbers of recipient mice per condition 2 weeks before the islet extraction at 11 weeks of age were as follows: 0, n = 5; 1, n = 5; 3, n = 6; 5, n = 6, and for 10, n = 5). The mRNA expression of *cdk11* in the PECs was measured by qRT-PCR. All comparisons were related to TSC: 0. A linear regression was performed according to the equation y=-0.036367x+1.106, resulting in p = 0.0177. **(E)** The percentages of the proliferating beta cells (CD45^-^ Glut2^+^ Ki67^+^) were measured by flow cytometry. Pancreatic islets were isolated from 11-week-old NOD (n = 6) and NOD/SCID (n = 4) mice, trypsinized and stained for CD45, Glut2, and Ki67). The data are represented as the mean ± SEM in **(A**, **E)**; in **(D)**, individual values are plotted. In **(A)** and **(E)**, the value of the NOD/SCID group was used as a reference. *p ≤ 0.05, **p ≤ 0.01.

At 11 weeks of age, the NOD female pancreatic islets are heavily infiltrated, while those from age-matched NOD/SCID female mice are insulitis-free, because they lack lymphocytes (36, 37). Using the cDNA microarray technology, we observed that the mRNA expression levels of *Cdk11* were 2.4-fold lower in the PECs from the 11-week-old pre-diabetic NOD female mice compared to those from age-matched NOD/SCID female mice (see **Supplementary Table 1**). We confirmed the microarray results by performing quantitative real time PCR (qRT-PCR) assays (**Figure 1A**) and found that the PECs from the 11-week-old NOD females exhibited a threefold reduction in the mRNA expression levels of *Cdk11* compared to those from age-matched NOD/SCID females (0.223 ± 0.047 vs. 0.674 ± 0.09, respectively). CDK11^p130^ protein expression was also reduced in PECs from NOD mice (**Figures 1B, C**). Nevertheless, CDK11^p58^ protein levels were higher in NOD PECs compared to those from NOD/SCID, underscoring the role of inflammation in post-transcriptional regulation of CDK11 (**Figures 1B, C**).

We further aimed to determine whether the mRNA expression of *Cdk11* was inversely correlated with the severity of the leukocyte infiltration into the islets. Therefore, we performed qRT-PCR to detect *Cdk11* in the PECs of the NOD/SCID islets from the 11-week-old female mice that had been adoptively transferred with increasing amounts of total leukocytes from prediabetic NOD female donors 2 weeks before the islet isolation. We observed a significant inverse relationship between *Cdk11* expression levels in PECs and the amount of transferred leukocytes (**Figure 1D**).

To address whether the downregulation of *Cdk11* mRNA prior to T1D onset altered the NOD beta cell replicative activity, we assessed the beta cell proliferation rates using Ki67 staining of GLUT-2^+^ CD45^-^ (beta) cells from 11-week-old NOD and NOD/SCID mice and analyzed these rates by flow cytometry (**Figure 1E**). We found no differences in the beta cell proliferation rates between the groups.

### CDK11 Hemideficiency Protects N-HTZ Mice Against Diabetes Without Affecting the Diabetogenicity of the Autoimmune Repertoire

**Figure 2:**
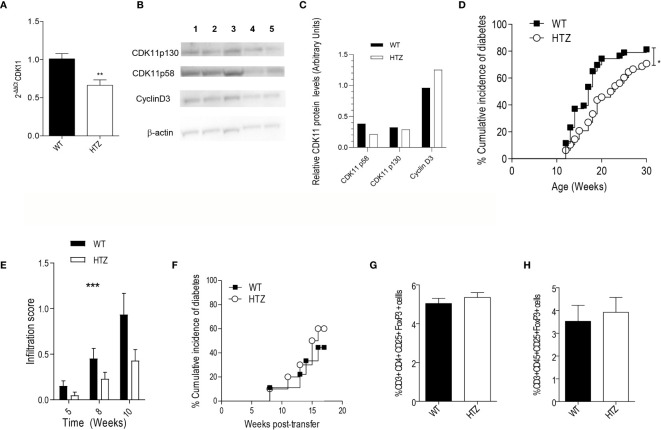
CDK11 hemideficiency protects against the onset of T1D without altering the diabetogenicity of the autoimmune repertoire. **(A)** Pancreatic islet cells were isolated from 6-week-old NS-WT and NS-HTZ (n = 5) mice. qRT-PCR analysis was performed to assess the mRNA expression of *cdk11*. **(B)** Pancreatic islet cells were isolated from 35-week-old NS-WT and NS-HTZ mice, each lane contains protein corresponding to pooled islets from two mice (130 islets per lane). Western blot analysis was performed to detect CDK11^p58^, CDK11^p130^, cyclin D3. **(C)** CDK11^p58^, CDK11^p130^, and Cyclin D3 expression values were respectively normalized to β-actin levels in pancreatic islets from either NS-WT (black columns) or NS-HTZ (white columns). The cumulative incidence of spontaneous diabetes in N-WT (n = 43) and N-HTZ (n = 48) mice was assessed. **(E)** Pancreata from 5-, 8-, and 10-week-old mice (minimum of n = 9 mice and maximum n = 16 per experimental group) were extracted, and the leukocyte infiltration levels were scored. **(F)** 4- to 5-week-old NOD/SCID recipient (n = 9 recipient mice for N-WT leukocyte donors; and n = 10 recipient mice for N-HTZ leukocyte donors) mice were adoptively transferred with 10 million of total cells from pancreatic lymph nodes (PLNs) from either 9-week-old N-WT or N-HTZ donors. The cumulative incidence of adoptively transferred diabetes is plotted. **(G)** Percentage of T regulatory lymphocytes (CD3^+^CD4^+^CD25^+^FoxP3^+^) in the PLNs (n = 12) is plotted. **(H)** Percentage of T regulatory lymphocytes (CD3^+^CD45^+^CD25^+^FoxP3^+^) in the lymphocytic infiltrate of the pancreatic islets [N-WT (n = 7) and N-HTZ (n = 6)] is shown. In **(G)** and **(H)**, 7-week-old N-WT and N-HTZ mice were used. The data are shown as the mean ± SEM in **(A**, **E**, **G**, **H)**; as the percentages of cumulative diabetic mice at a particular time point in **(D**, **F)**. *p ≤ 0.05, **p ≤ 0.01, ***p ≤ 0.001.

To ascertain whether the mRNA downregulation of *Cdk11* at the onset of T1D was causally related to beta cell death *in vivo*, we developed a NOD mouse model that was genetically hemideficient in CDK11 (N-HTZ) (**Figure 2A**), since the homozygous CDK11 deficiency is embryonically lethal (22). We also obtained NOD/SCID mice that were hemideficient in CDK11 (NS-HTZ) and checked protein expression levels in NS-HTZ versus NS-WT islet cells. CDK11^p58^ protein levels were reduced while CDK11^p130^ reduction was minimal in NS-HTZ compared to NS-WT mice (**Figures 2B, C**). This outcome revealed a differential translational regulation in CDK11 hemideficency favoring CDK11^p130^ over CDK11^p58^.

We monitored the spontaneous onset of T1D in the N-HTZ and N-WT littermate mice. In parallel we assessed the spontaneous incidence of diabetes in NS-HTZ compared to that in NS-WT littermates, in case an intrinsic defect in beta cell generation was related to CDK11 hemideficiency.

We found that the N-HTZ mice exhibited a significantly delayed kinetics of the disease and lower cumulative incidence of diabetes (70.8% vs. 81.4% respectively) compared to the N-WT littermates (**Figure 2D**). None of the NS-HTZ mice developed spontaneous diabetes (see **Supplementary Table 2**), which evidenced that no major intrinsic defect in beta cell mass and/or function is associated to the CDK11 hemideficiency.

To determine whether the protection against T1D shown by the N-HTZ mice was due to a milder insulitic attack compared to the N-WT mice, we first scored the islet leukocyte infiltration associated with both genotypes at three different time points prior to the onset of diabetes (5, 8, and 10 weeks of age). Dramatic differences were found in the severity of the insulitic attack between both genotypes independently of the age tested, being the infiltration score significantly lower in the N-HTZ genotype compared to the N-WT (**Figure 2E**).

We further assessed the diabetogenicity of the N-HTZ leukocyte repertoire compared to that of N-WT littermates by performing adoptive transfer of diabetes into NOD/SCID recipients. In total, 10 million cells isolated from Pancreatic Lymph Nodes (PLNs) from either 9-week-old N-HTZ or N-WT donors were transferred into NOD/SCID recipient mice (**Figure 2F**). The onset of the adoptively transferred diabetes was monitored, and no difference between both genotypes was observed in their diabetogenic capacity, which was supported by the unaltered representation of Tregs in the PLNs and insulitic infiltrate from N-HTZ females compared to the N-WT genotype (**Figures 2G, H** respectively). We also evaluated the frequency of effector and naïve T cells in PLNs and in the insulitic infiltrates; the *in vitro* proliferative activity and apoptotic rates of PLN cells in front of stimulation by islet autoantigens (**Supplementary Figure 1**); and no major difference was found between both genotypes. Moreover, *in vitro* Th1 response was not impaired in PLN cells from N-HTZ mice (**Supplementary Figure 2**).

Therefore, the target tissue itself, the beta cell compartment, and no an impaired autoimmune repertoire in the N-HTZ mice, should account for the protection against diabetes exerted by the CDK11 hemideficiency. NOD macrophages are less efficient in engulfing apoptotic cells leading to inflammation (38). The release of smaller amounts of beta cell antigens by N-HTZ islets due the lower apoptosis rates, would not sustain islet inflammation as is fostered by the inefficient scavenger capability of N-WT macrophages, leading, hence, to a lower infiltration score in N-HTZ.

### CDK11 Promotes Cytokine-Induced Beta Cell Apoptosis

**Figure 3:**
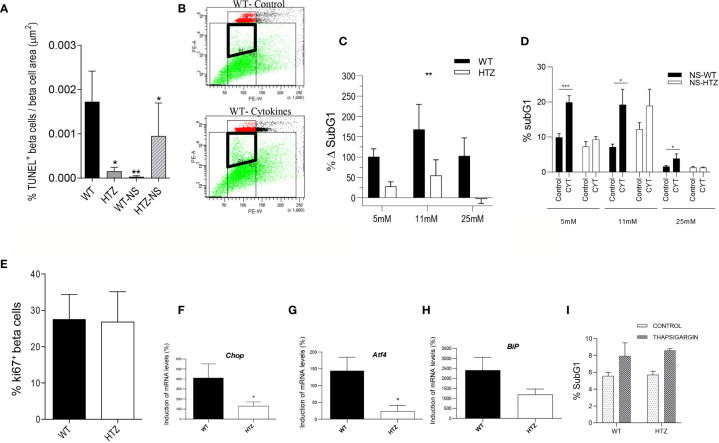
CDK11 hemideficiency protects beta cells against inflammation-induced apoptosis. **(A)** TUNEL assessment of apoptotic beta cells was performed in pancreatic sections from 5-week-old N-WT, N-HTZ, NS-WT and NS-HTZ mice (n = 7 mice per genotype; N-WT: 275 islets, N-HTZ: 237 islets, NS-WT: 201 islets, NS-HTZ 232 islets) and quantified using epifluorescence microscopy. TUNEL-positive nuclei per insulin-positive area were measured in each experimental group. **(B**–**D)** Induction of DNA fragmentation as a measurement of apoptosis induction by proinflammatory cytokines. NS-WT and NS-HTZ pancreatic islets (25 islets per condition) from 5-week-old female mice were cultured either in the presence or absence of cytokines (IFN-γ + TNF-α + IL1β) (n = 7 experiments) for 24 hours at the indicated glucose concentrations, then, cells were harvested and stained to quantify DNA fragmentation by Flow Cytometry. **(B)** Gating strategy is presented: Gate P1 comprehends the apoptotic SubG1 events used for subsequent calculations. Both panels correspond to NS-WT islets cultured in 5mM glucose for 24 h, in the presence (bottom panel) or absence (upper panel) cytokines. Results are plotted either as percentage of increment in SubG1 events upon cytokine exposure **(C)** or raw data comparing basal levels of subG1 to those after cytokine exposure **(D)**. **(E)** Islet cells from 6-to-8-week-old NS-WT and NS-HTZ (n = 12) mice were stained to assess the beta cell proliferative activity; the percentages of CD45^-^ Glut2^+^ Ki67^+^ proliferating cells are plotted from one experiment that is representative of three independent experiments. **(F**–**H)** Upregulation of UPR markers upon ER-stress induction. NS-WT and NS-HTZ pancreatic islets (50 islets per conditon) from 5–7-week-old female mice were cultured for 24 h either in the presence or absence of 1 μM Thapsigargin, harvested for RNA isolation and qRT-PCR of *Chop* **(F)**, *Atf4* **(G)**, and *BiP* **(H)** was performed. The graphs plot the induction of RNA levels compared to those of untreated controls. Data are means samples ± SEM (n = 4 independent experiments). **(I)** DNA fragmentation (%subG1) as a measurement of apoptosis induction by thapsigargin. NS-WT (n = 4 mice) and NS-HTZ (n = 3 mice) pancreatic islets (30 islets per condition) from 5-week-old female mice were cultured for 96 h either in the presence or absence of thapsigargin (1µM). ***p < 0.005, **p < 0.01, and *p < 0.05.

We further explored the relationship between the viability of beta cells and the expression levels of CDK11. We determined the number of late apoptotic beta cells using the TUNEL assay in WT and HTZ mice from both 5-week-old NOD and NOD/SCID strains (**Figure 3A**) (**Suplementary Figure 3**). In the NOD strain, the N-WT beta cells exhibited a dramatic increase (10.8 times) in apoptotic activity compared to those from N-HTZ (173 × 10^-5^ ± 69 × 10^-5^ vs. 16 × 10^-5^ ± 9 × 10^-5^, respectively). The differences in the apoptosis rates between the WT and HTZ genotypes were abrogated in the NOD/SCID strain (3 × 10^-5^ ± 2 × 10^-5^ vs. 95 ± 74 × 10^-5^, respectively) (**Figure 3A**). These results reveal that the protection against T1D exerted by the CDK11 hemideficiency in NOD mice is caused by a negative interference with cytokine (inflammation)-induced apoptosis, since, in the NOD/SCID strain, as expected, the apoptosis levels are negligible regardless of CDK11 expression levels, because there is no inflammation.

Altogether, these observations suggested a scenario in which the inflammation-driven cleavage of CDK11^p58^ and CDK^p130^ into CDK11^p46^ and, the induction of CDK11^p58^ in a mitotic effort to compensate for the initial beta cell loss induced by inflammation in T1D (30), would enhance beta cell apoptosis. Therefore, the physiological downregulation of CDK11 should have a protective effect against T1D onset.

In order to confirm the hypothesis that CDK11 hemideficiency protects from cytokine-induced apoptosis, we cultured pancreatic islets from either wild type (NS-WT) or CDK11 hemideficient (NS-HTZ) NODSCID female mice in the presence of a proinflammatory cytokine cocktail (TNFα+IL-1β+IFNγ) at either low (5mM), medium (11mM) or high (25mM) glucose concentration, respectively, for 24 h. Subsequently, the induction of DNA fragmentation was assessed by flow cytometry as a measurement of late apoptotic events (**Figures 3B–D**). The induction of apoptosis due to the exposure to pro-inflammatory cytokines was significantly lower in the NS-HTZ group compared to the NS-WT control group, independently of the glucose concentration. Therefore, an impairment in the pro-inflammatory trigger of apoptosis does account for the protection exerted by the CDK11 hemideficiency against beta cell apoptosis. This finding highlighted the role of CDK11 in promoting apoptosis once the proinflammatory milieu pervades the pancreatic islets during the progression of the autoimmune attack.

Since CDK11^p58^ has been proven to be essential for sister chromatid cohesion (18, 19), we investigated whether the hemideficiency in the CDK11 resulted in impaired beta cell proliferation too. The percentage of proliferating beta cells (CD45^-^Glut-2^+^Ki67^+^) was assessed by flow cytometry (**Figure 3E**), but, surprisingly, no differences were found in the beta cell proliferation rate between the genotypes, which implied that the role that CDK11 plays in beta cell viability is cell-cycle-independent.

### CDK11 Is Involved in the Upregulation of Atf4 and Chop in Beta Cell Response to ER-Stress, Although ER-Stress Induced Apoptosis Is Not Altered by CDK11 Hemideficiency

In order to ascertain whether CDK11 hemideficiency also inhibits UPR-induced apoptosis in beta cells, we cultured pancreatic islets from either wild type (NS-WT) or CDK11 hemideficient (NS-HTZ) NODSCID female mice in the presence or absence of Thapsigargin, a drug that induces ER-stress. 24 h later, the islets were collected and the RNAs of all experimental groups extracted to quantify the relative expression levels of three key UPR markers: *BiP* (Binding-immunoglobulin protein, a chaperone) (31); *Atf4* (Activating transcription Factor 4) (39); and *Chop* (C/EBP Homologous Protein) (32). ATF4 leads to CHOP expression, which leads to apoptosis in terminal UPR.

The results obtained showed a significant reduction in the upregulation of both, *Chop* (132.1 ± 39.61 vs. 411.6 ± 140.4) (**Figure 3F**) and *Atf4* (23.79 ± 17.51 vs. 144.2 ± 40.17) (**Figure 3G**), upon ER-stress induction in the NS-HTZ compared to the NS-WT islets, respectively. In NS-HTZ, the induction of both transcription factors by ER-stress is severely impaired. However, ER-stress-induced *BiP* upregulation, was not significantly different between the NS-HTZ genotype compared to the NS-WT islets (1191 ± 277,4 vs. 2407 ± 647,4, respectively) (see **Figure 3H**).

This outcome evidenced that CDK11 could have a role in promoting UPR-induced beta cell death, since CDK11 hemideficiency would protect beta cells from apoptosis by inhibiting UPR-induced CHOP expression. To further assess this hypothesis, we cultured pancreatic islets from both, NS-WT and NS-HTZ in either the presence or absence of Thapsigargin for 96 h (4 days), collected the islets, and measured %subG1 events by flow cytometry (**Figure 3I**). Surprisingly, no differences in the levels of Thasigargin-induced apoptosis were observed between both genotypes. This outcome revealed that CDK11 hemideficiency had not a significant role in inhibiting ER-stress induced apoptosis in beta cells.

### Cyclin D3 Promotes the Viability of Beta Cells Independently of CDK11 Signaling

*Cdk11*, similarly to Cyclin D3, is downregulated in beta cells following an autoimmune attack in T1D. However, while the downregulation of Cyclin D3 compromises the beta cell viability, the downregulation of CDK11 is protective against beta cell death. This is an intriguing finding since Cyclin D3 is a regulatory partner of CDK11^p58^ (40–44), which, in turn, is required for cell cycle progression and is involved in apoptosis. Therefore, in the absence of Cyclin D3, CDK11^p58^-promoted apoptosis should be impaired, and NOD mice that are deficient in Cyclin D3 should exhibit milder T1D due to its role in activating CDK11^p58^. However, we previously showed (10) that the Cyclin D3 deficiency dramatically exacerbates T1D in NOD mice, while its overexpression in beta cells protects them against diabetes; furthermore, neither of these conditions, i.e., deficiency or overexpression, has any effect on beta cell replication (10), which strongly suggests that the role of Cyclin D3 in beta cells is independent of CDK11^p58^.

Thus, we speculated about the relationship between CDK11 and Cyclin D3 regarding beta cell viability and whether a hierarchy exists among the respective signaling pathways that are engaged by these two molecules in beta cells. Our working hypothesis is that CDK11 may work as a decoy receptor for Cyclin D3, quenching Cyclin D3 anti-apoptotic activity in beta cells. In the N-HTZ genotype, the levels of free, unbound Cyclin D3 are higher than in N-WT, and hence, beta cell survival is promoted. Therefore, if this was the case, we would not expect any amelioration of the severity of the disease in Cyclin D3 deficient mice upon CDK11 hemideficiency.

We answered this question by obtaining NOD mice deficient in Cyclin D3 that were either hemideficent (K11HTZ-D3KO) or not (K11WT-D3KO) for CDK11.

**Figure 4:**
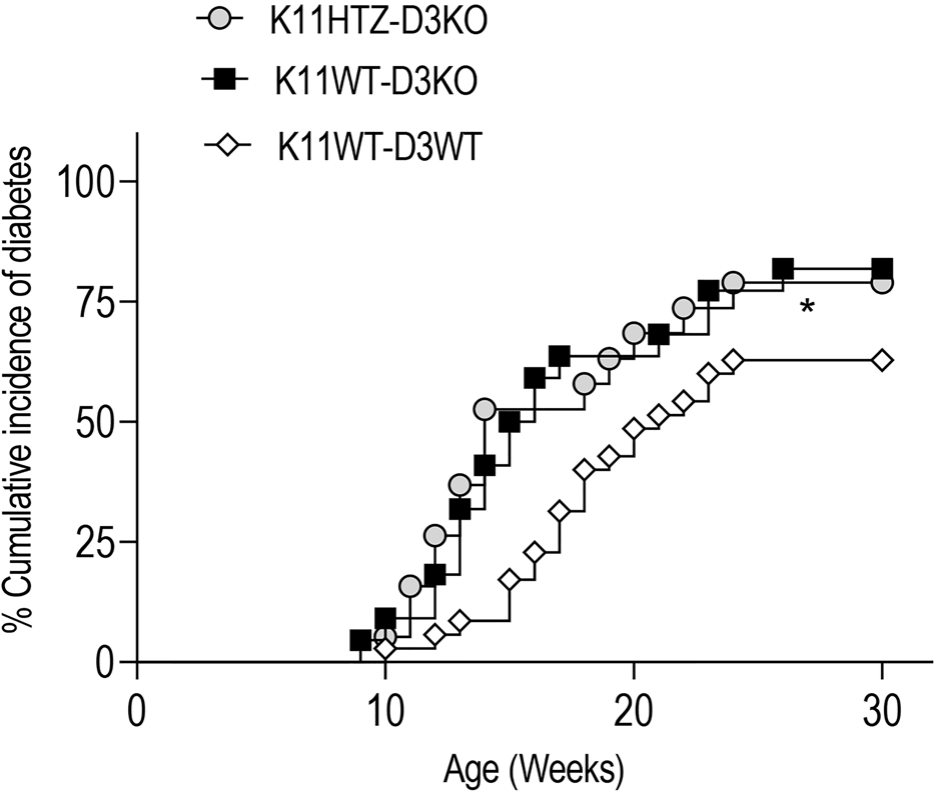
CDK11 hemideficiency protection against T1D depends on Cyclin D3 availabilty. The cumulative diabetes incidence in K11HTZ-D3KO (n = 19), K11WT-D3KO (n = 23) and K11WT-D3WT (n = 34) mice was assessed. The data are shown as the percentages of the cumulative diabetic mice at a particular time point. *p ≤ 0.05.

We monitored the spontaneous incidence of diabetes in these two experimental groups and observed that regardless of the CDK11 hemideficiency, the Cyclin D3 deficiency had a dominant effect on the onset of diabetes and exacerbated the disease compared to normal NOD mice (K11WT-D3WT) since the CDK11 hemideficiency did not alleviate the T1D in the Cyclin D3 deficient mice (**Figure 4**).

Altogether, this observation suggested that at least one of the signaling pathways responsible for the survival of beta cells in which Cyclin D3 is involved is independent of the CDK11 signaling pathway.

## Discussion

### Role of CDK11 in the Beta Cell Compartment

CDK11 is a pleiotropic kinase that is involved in gene expression, cell division and cell death. The function of CDK11 and its regulation in these processes remain unclear in beta cells. Beta cells have been shown to increase their proliferation rates prior to the onset of T1D (30), therefore, it is plausible that while the overall mRNA expression of *Cdk11* is downregulated due to insulitis, the translation of CDK11^p58^ is favored at the expense of CDK11^p130^ in an attempt to subdue the loss of beta cells. However, this defense mechanism may be detrimental to beta cell survival because CDK11^p58^ also signals for mitosis-coupled apoptosis (24) and becomes a substrate for the amplification of caspase-3, which would result in cytokine-induced beta cell death in T1D. Therefore, downregulation of Cdk11mRNA would prevent massive CDK11^p58^ translation under inflammatory circumstances. In the spinal cord and astrocytes, the CDK11^p58^ protein is also upregulated during inflammation. Moreover, in Schwann cells, a pro-inflammatory stimulus, such as LPS, could upregulate CDK11^p58^, and the partnership CDK11^p58^/Cyclin D3 inhibits of the cell cycle and promotes apoptosis (43). In this sense we found that the increased CDK11^p58^ expression levels in NOD PECs prior to the onset of diabetes did not alter beta cell replication. Moreover, genetic CDK11 hemideficiency, (affecting mostly to the CDK11^p58^ protein compartment, while CDK11^p130^ remains roughly untouched in endocrine cells), protected beta cells from apoptosis in the NOD proinflammatory niche. Furthermore, when exposed to a powerful proinflammatory mileu NS-HTZ islets exhibited significantly lower apoptotic rates compared to those from the NS-WT control group, confirming the involvement of CDK11^p58^ in cytokine-induced apoptosis. Therefore, the scenario in which CDK11 would be cleaved upon inflammation promoting apoptosis with a dominant effect over the other CDK11 roles, is evidenced as a mechanism responsible for protection against beta cell apoptosis in the CDK11 hemideficient NOD mice.

ER-stress and inflammation are responsible for most of the beta cell failure in T1D. Both, inflammation and ER-stress crosstalk and feed-back each other positively, resulting in exacerbation of beta cell death (33). It has been reported that interfering the UPR signaling reverses T1D in the NOD mouse model (45). Both, CDK11^p58^ (24) and, CHOP (32), a molecule causally related to ER-stress-induced apoptosis, have been reported to promote apoptosis by interfering with the expression of Bcl-2, a key anti-apoptotic molecule in ER-stress (46). However, we have not observed an impairment in the induction of apoptosis by ER-stress associated to the CDK11 hemideficiency, which suggests that other (not dependent on CHOP) apoptotic cascades in terminal UPR, such as those downstream IRE1 (47), remain unaltered, and CDK11 would be mainly involved in cytokine-induced apoptosis.

The expression of the CDK11 promoter is hindered by inflammation, probably through Ets-1 transcription factor (48–51), resulting in a protection against apoptosis in beta cells. In the other cell types, cyclin D3 interacts with CDK11^p58^ to promote cell division and/or apoptosis. In adult mouse beta cells, we did not observe changes in replication due to either Cyclin D3 or the CDK11 hemideficiency, which suggests that the protection against diabetes is not related to proliferation. Moreover, *a priori*, one would expect that the deficiency in Cyclin D3, which is a putative activator of CDK11^p58^, should promote a phenotype that is similar to that observed in the CDK11 hemideficient mice, and the deficiency of both should add up to the same direction. However, this is not the case since both molecules have opposite effects on the onset of T1D. This observation shows that the downregulation of CDK11 exerts its protection against T1D in a Cyclin D3-dependent fashion. This outcome would be compatible with a scenario in which CDK11^p58^ binds Cyclin D3 to prevent its signaling to promote beta cell survival, and, to activate CDK11^p58^ pro-apoptotic activity. Therefore, the anti-apoptotic role of Cyclin D3 should be more prominent in non-cycling cells, a scenario when CDK11^p58^ is not expressed.

### Role of CDK11 in the Autoimmune Repertoire

The diabetogenicity of the autoimmune repertoire was not affected in N-HTZ mice. Moreover, the representation of the FoxP3^+^CD25^+^CD4^+^ Treg cell subset in either the regional lymph nodes or the islet infiltrate in not altered in CDK11 hemideficient mice. Therefore, the protection observed against T1D in N-HTZ cannot be explained by an altered diabetogenic repertoire in this genotype.

## Conclusion

CDK11 downregulation prior to T1D onset has a protective effect on beta cell survival in an inflammatory context. This protective effect depends on Cyclin D3 availability. Further work needs to be performed to identify the relationship between CDK11-mediated proapoptotic signaling and Cyclin D3 in beta cells, to design better therapeutic approaches targeting T1D.

## Data Availability Statement

The original contributions presented in the study are included in the article/**Supplementary Material**. Further inquiries can be directed to the corresponding author.

## Ethics Statement

The animal study was reviewed and approved by the Institutional Ethical Committee for Animal Experimentation of the University of Lleida (CEEA).

## Author Contributions

CM designed the study. ES, CV, JL, NS-A, US, LH, SV-P, JP, and CM performed the study. TS, JV, AC, AR, AN, and MV-C contributed new reagents/analytic tools and revised the manuscript. JML provided the CDK11 hemideficient mouse model in the mixed 129SvxC57BL/6 genetic background. CM wrote the paper and supervised the study. All authors contributed to the article and approved the submitted version.

## Funding

This work was supported by the Spanish Ministerio de Economía, Industria y Competitividad Grant SAF2017-82567-R, Ministerio de Ciencia y Tecnología Grant SAF2008-02536, and, Ministerio de Ciencia, Innovación y Universidades Grant SAF2014-55077-R (to CM) and SAF2016-77227-R and SAF2013-45140-R (to TS and JV); the 2009 SGR-DGR grant from the Generalitat de Catalunya (to JV and CM) and the TR265 no. A001E-12132/2009 from the University of Lleida (to JV and CM); the Generalitat Valenciana PROMETEO/2011/080 grant; and the Ministerio de Economía y Competitividad BFU2011-28358 grant (to AN). NS-A and CV were recipients of a predoctoral fellowship from the University of Lleida. US, ES, and JL have been granted with AGAUR predoctoral fellowships from the Generalitat de Catalunya. CM and JV are assistant professors of Immunology in the Serra Hunter Program at the University of Lleida and, investigators at the same institution and the Lleida Institute for Biomedical Research Fundación Pifarré.

## Conflict of Interest

The authors declare that the research was conducted in the absence of any commercial or financial relationships that could be construed as a potential conflict of interest.

The reviewer XD declared a shared affiliation, with no collaboration, with one of the authors, JL, to the handling editor at the time of review.

## Abbreviations

CDK11, Cyclin dependent Kinase 11; T1D, Type 1 Diabetes; PEC, Pancreatic Endocrine Cells; NOD, Non Obese Diabetic; qRT-PCR, quantitative Real Time-Polymerase Chain Reaction; WT, Wild Type; HTZ, Heterozygous; UPR, Unfolded Protein response; ER, Endoplasmic Reticulum; *Bip*, Binding-immunoglobulin protein; *Atf4*, Activating Transcription Factor 4; *Chop*, C/EBP Homologous Protein.
