# Supplementary material for: CDK11 Promotes Cytokine-Induced Apoptosis in Pancreatic Beta Cells Independently of Glucose Concentration and Is Regulated by Inflammation in the NOD Mouse Model
Source: Front Immunol. 2021 Feb 10;12:634797. doi: 10.3389/fimmu.2021.634797 (PMC7923961; doi:10.3389/fimmu.2021.634797)
Supplement: Supplementary file 1 [file DataSheet_1.docx]

**SUPPLEMENTARY METHODS**

**Quantitative real time PCR (qRT-PCR)**

**cdk11 mRNA relative levels.** Total RNA was extracted from NODSCID PECs using TRIzol reagent (Invitrogen, Carlsbad, CA, USA), and 500 ng of the total RNA in 20 μL was reverse-transcribed into cDNA using the iScript cDNA Synthesis Kit (Bio-Rad, Hercules, CA, USA). The qRT-PCR mixture contained 5 μl of iTaq Universal SYBR Green Supermix (Bio-RAD), 4 μl of cDNA (diluted 1:5), and 0.5 μl of each of the forward and reverse primers in a final volume of 10 μl. The primers used for the detection of mouse cdk11 were mcdk11BFwRT- TCTGCACATCACCGTACCAT and mcdk11BRvRT-CTGGTTTTCCTCTCGCTGTC; and those for β-actin were β-actinFw-TGGAATCCTGTGGCATCCATGAAA and β-actinRev-TAAAACGCAGCTCAGTAAGAGTCC. The qRT-PCR procedure was performed as follows: 95°C for 3 min; 95°C for 10 s, 63°C for 40 cycles; and a melting curve from 65 to 95°C; the qRT-PCR was performed on a CFX 96 Real time system (Bio-Rad, USA) and analyzed using Software CFX manager 3.1 version (Bio-Rad, USA). β-actin was used as a control to normalize the gene expression. The data obtained were calculated using the 2−ΔΔCt method.

**UPR markers (BiP, ATF4 and CHOP) mRNA relative levels.** One microgram of RNA was reverse transcribed and quantitative gene expression was evaluated by qPCR performed on a BioRad MiniOpticonTM Real-Time PCR system. The data obtained were calculated using the 2−ΔΔCt method, and expressed relative to the expression of the housekeeping gen *Gadph*. (Thermal cycling conditions were as follows: activation of Taq DNA polymerase at 95ºC for 10 minutes, followed by 40 cycles of amplification at 95ºC for 15 seconds and at 60ºC for 1 minute). Induction level is expressed as % as follows:

[(relative expression levels with Thapsigargin treatment) – (relative expression levels in control conditions)]x100

(relative expression levels in control conditions)

The primer sequences used for qRT-PCR are as follows [35, 36]: mBip Fw CAGATCTTCTCCACGGCTTC, mBip Rv GCAGGAGGAATTCCAGTCAG; mChop Fw CGAAGAGGAAGAATCAAAAACCTT, mChop Rv GCCCTGGCTCCTCTGTCA; mAtf4 Fw AGCAAAACAAGACAGCAGCC. mAtf4 Rv ACTCTCTTCTTCCCCCTTGC; mgadph Fw TGTGTCCGTCGTGGATCTGA, mgadph Rv CCTGCTTCACCACCTTCTTGA.

**Western blot assays**

Islet and PEC lysates were obtained using MPER lysis buffer supplemented with HALT protease inhibitor cocktail (Pierce, Rockford, IL, USA). Anti-CDK11 antibodies (SC-928, Santa Cruz Biotechnology, Dallas, TX, USA); PIC rabbit polyclonal provided by J. Lahti) were used to quantify CDK11 protein expression levels, anti-Cyclin D3 antibody was used to detect Cyclin D3 (DCS-22, BioLegend, San Diego, CA, USA); all results were normalized to β-actin levels (house-keeping) with anti-β-actin antibody (AC-74 Sigma Aldrich). Image acquisition and band quantification was performed using the Image Lab software (Bio-Rad).

**Assessment of immune cell subsets by flow cytometry**

Single cell suspensions were obtained from pancreatic lymph nodes (PLN) and islet infiltrating leukocytes (IILs) from wild-type (N-WT) or CDK11 hemideficient (N-HTZ) NOD mice. The isolated cells were stained to assess the surface expression of CD3, CD4, CD25 (BD), and the intracellular expression of FoxP3 (eBioscience, Life Technologies, Carlsbad, CA)) by flow cytometry.

**Cell culture of lymphocytes** was performed in RPMI 1640 media (Lonza) supplemented with 10% FBS, 1.21 mM gentamicin, and 0.2 mM beta-mercaptoethanol in a humidified atmosphere of 5% CO2 at 37°C.

**T cell proliferation and Annexin V staining**

Single cell suspensions were obtained from the spleen and PLN of 7-week-old mice. The cells were labeled with CFSE (Molecular Probes, Eugene, Oregon, US) according to the manufacturer’s instructions and cultured in 12-well plates (1×106 cells/1 ml per well) either in the presence of 1 μg/ml of the agonistic anti-CD3 Ab (2C11 clone) (BD) or with 25 μg/ml of the following antigens: recombinant human insulin (rhINS) (Sigma-Aldrich), insulin B chain peptide (p12-25) (INSB12-25) (VEALYLVCGERGFF; Sigma-Genosys), glutamic acid decarboxylase 65 (GAD65) peptide (p35) (SRLSKVAPVIKARMMEYGTT; Sigma-Genosys) and keyhole limpet hemocyanin (KLH) (Sigma-Aldrich). The cells were incubated 72 hours prior to the proliferation assessment or 24 hours for the lymphocyte apoptosis quantification using Annexin V staining (BD) by Flow Cytometry.

**Secretion of Lymphocyte IL2 and INFγ**

Single cell suspensions were obtained from the spleen and PLN of 7-week-old mice and cultured in 12-well plates (1×10^6^ cells/1 ml per well) in the presence of 1 μg/ml of the agonistic anti-CD3 Ab (2C11 clone) for 48 hours. The cell culture supernatant was collected and IL-2 and IFNγ were measured using a standard sandwich ELISA (BD) and peroxidase OPD as a substrate (Sigma-Aldrich).
