# Supplementary material for: CDK11 Promotes Cytokine-Induced Apoptosis in Pancreatic Beta Cells Independently of Glucose Concentration and Is Regulated by Inflammation in the NOD Mouse Model
Source: Front Immunol. 2021 Feb 10;12:634797. doi: 10.3389/fimmu.2021.634797 (PMC7923961; doi:10.3389/fimmu.2021.634797)
Supplement: Supplementary file 2 [file Presentation_1.pptx]

## Slide 1
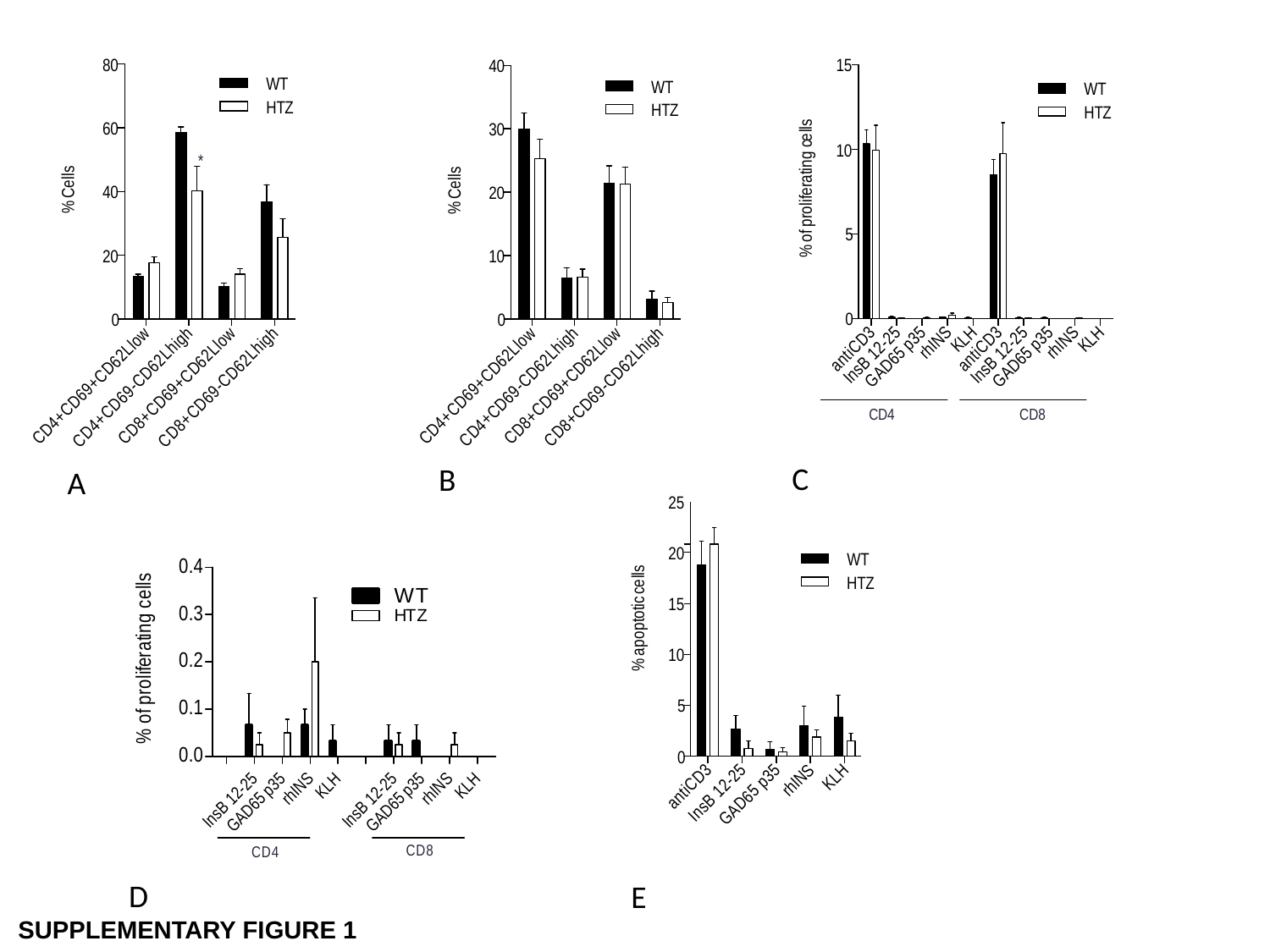

40
WT
HT
Z
30
s
l
l
e
C
20
%
10
0
h
h
w
w
g
g
o
o
i
i
l
l
h
h
L
L
L
L
2
2
2
2
6
6
6
6
D
D
D
D
C
C
C
C
+
+
-
-
9
9
9
9
6
6
6
6
D
D
D
D
C
C
C
C
+
+
+
+
4
8
4
8
D
D
D
D
C
C
C
C
15
WT
HT
Z
s
l
l
e
c
10
g
n
i
t
a
r
e
f
i
l
o
r
p
f
5
o
%
0
3
5
5
3
5
5
S
S
H
H
2
3
2
3
D
D
L
L
N
N
-
-
p
p
I
I
K
K
C
C
2
2
h
h
i
i
5
5
1
1
r
r
t
t
6
6
n
n
B
B
a
a
D
D
s
s
A
A
n
n
I
I
G
G
CD4
CD8
80
WT
HT
Z
60
*
s
l
l
e
C
40
%
20
0
h
h
w
w
g
g
o
o
i
i
l
l
h
h
L
L
L
L
2
2
2
2
6
6
6
6
D
D
D
D
C
C
C
C
+
+
-
-
9
9
9
9
6
6
6
6
D
D
D
D
C
C
C
C
+
+
+
+
4
8
4
8
D
D
D
D
C
C
C
C
C
B
A
25
20
WT
s
l
l
e
HT
Z
c
c
15
i
t
o
t
p
o
p
a
10
%
5
0
3
5
5
S
H
2
3
D
L
N
-
p
I
K
C
2
h
i
5
1
r
t
6
n
B
a
D
s
A
n
I
G
D
E
SUPPLEMENTARY FIGURE 1

## Slide 2
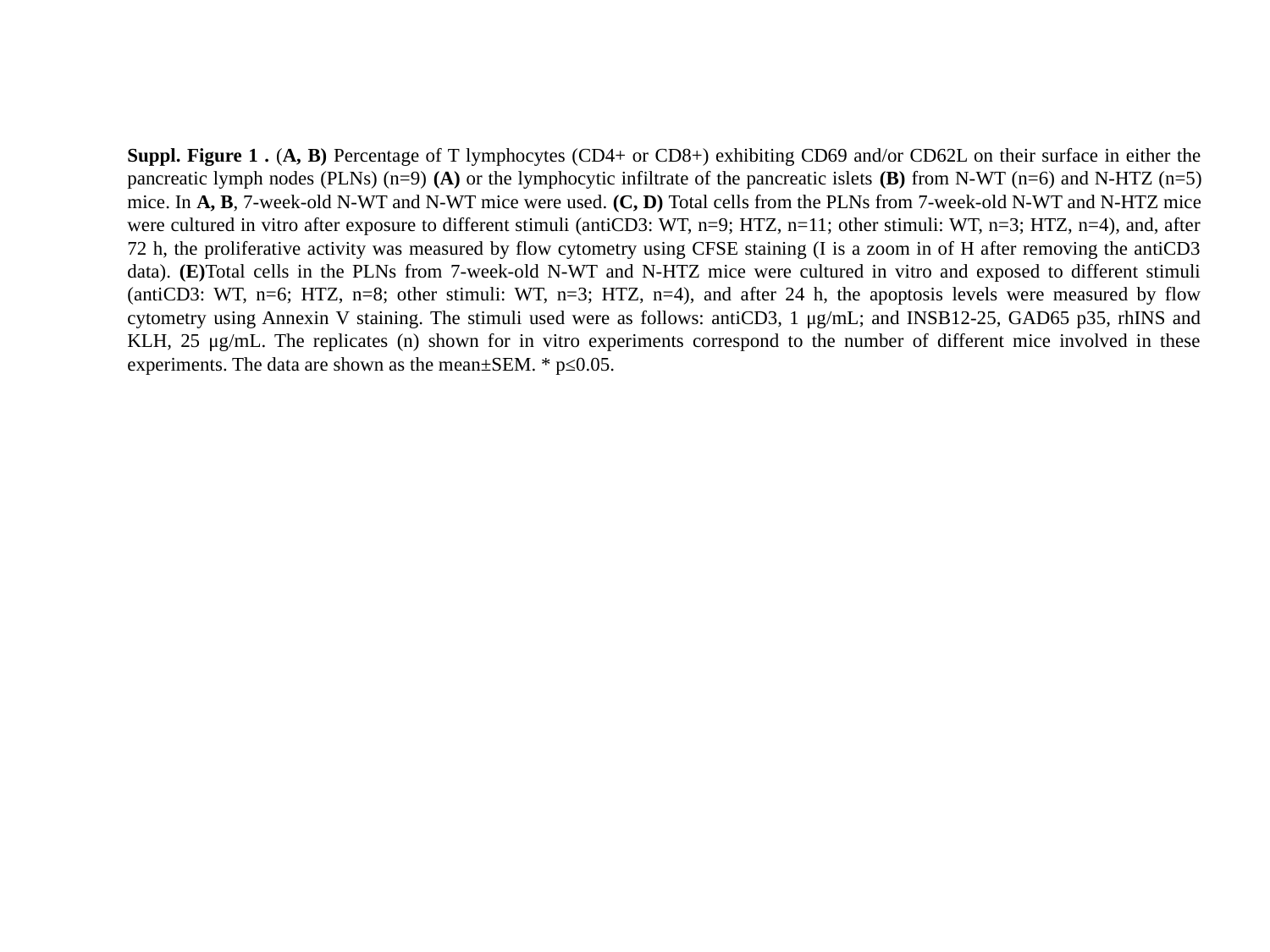

Suppl. Figure 1 . (A, B) Percentage of T lymphocytes (CD4+ or CD8+) exhibiting CD69 and/or CD62L on their surface in either the pancreatic lymph nodes (PLNs) (n=9) (A) or the lymphocytic infiltrate of the pancreatic islets (B) from N-WT (n=6) and N-HTZ (n=5) mice. In A, B, 7-week-old N-WT and N-WT mice were used. (C, D) Total cells from the PLNs from 7-week-old N-WT and N-HTZ mice were cultured in vitro after exposure to different stimuli (antiCD3: WT, n=9; HTZ, n=11; other stimuli: WT, n=3; HTZ, n=4), and, after 72 h, the proliferative activity was measured by flow cytometry using CFSE staining (I is a zoom in of H after removing the antiCD3 data). (E)Total cells in the PLNs from 7-week-old N-WT and N-HTZ mice were cultured in vitro and exposed to different stimuli (antiCD3: WT, n=6; HTZ, n=8; other stimuli: WT, n=3; HTZ, n=4), and after 24 h, the apoptosis levels were measured by flow cytometry using Annexin V staining. The stimuli used were as follows: antiCD3, 1 μg/mL; and INSB12-25, GAD65 p35, rhINS and KLH, 25 μg/mL. The replicates (n) shown for in vitro experiments correspond to the number of different mice involved in these experiments. The data are shown as the mean±SEM. * p≤0.05.

## Slide 3
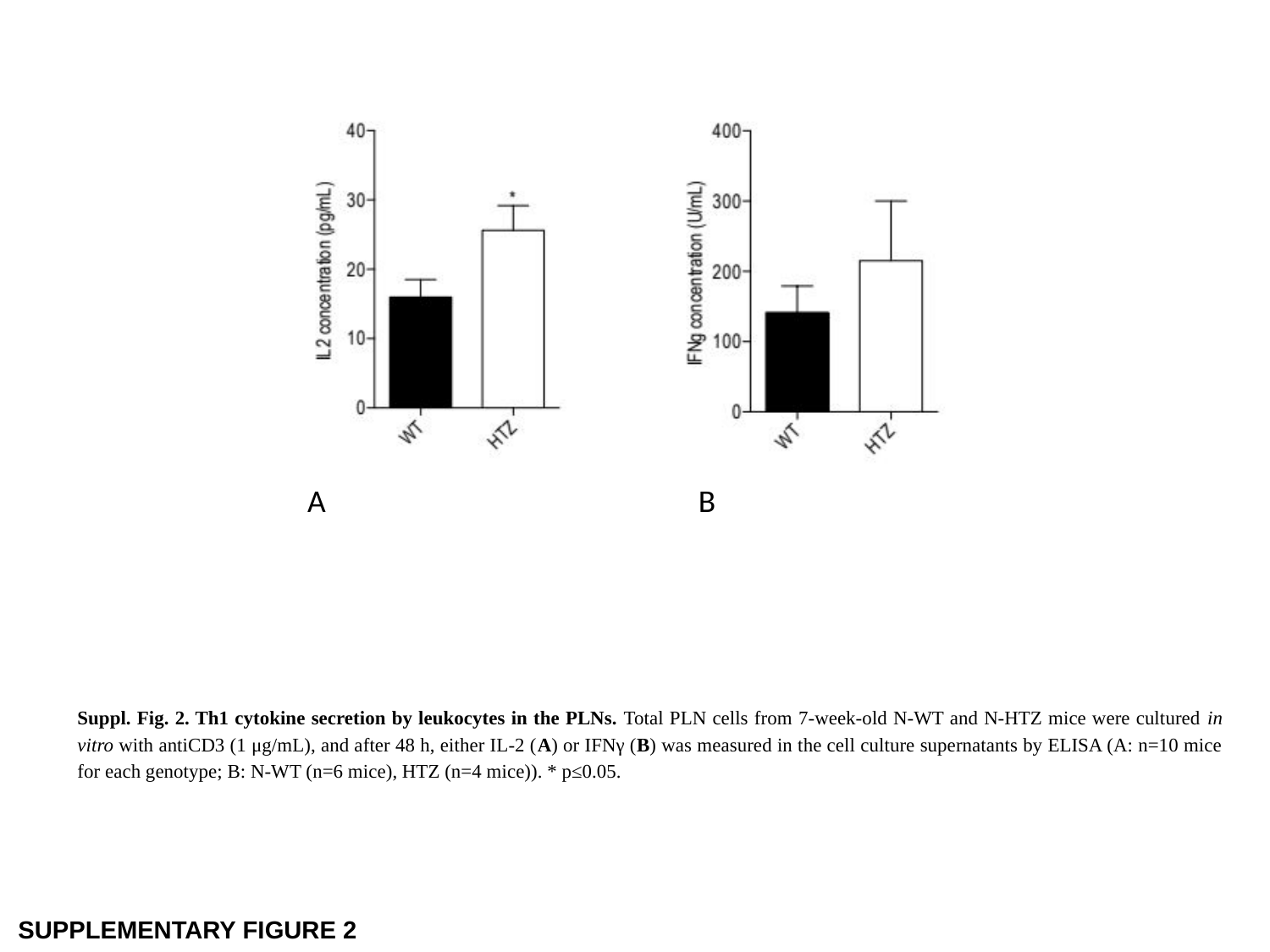

A
B
Suppl. Fig. 2. Th1 cytokine secretion by leukocytes in the PLNs. Total PLN cells from 7-week-old N-WT and N-HTZ mice were cultured in vitro with antiCD3 (1 μg/mL), and after 48 h, either IL-2 (A) or IFNγ (B) was measured in the cell culture supernatants by ELISA (A: n=10 mice for each genotype; B: N-WT (n=6 mice), HTZ (n=4 mice)). * p≤0.05.
SUPPLEMENTARY FIGURE 2

## Slide 4
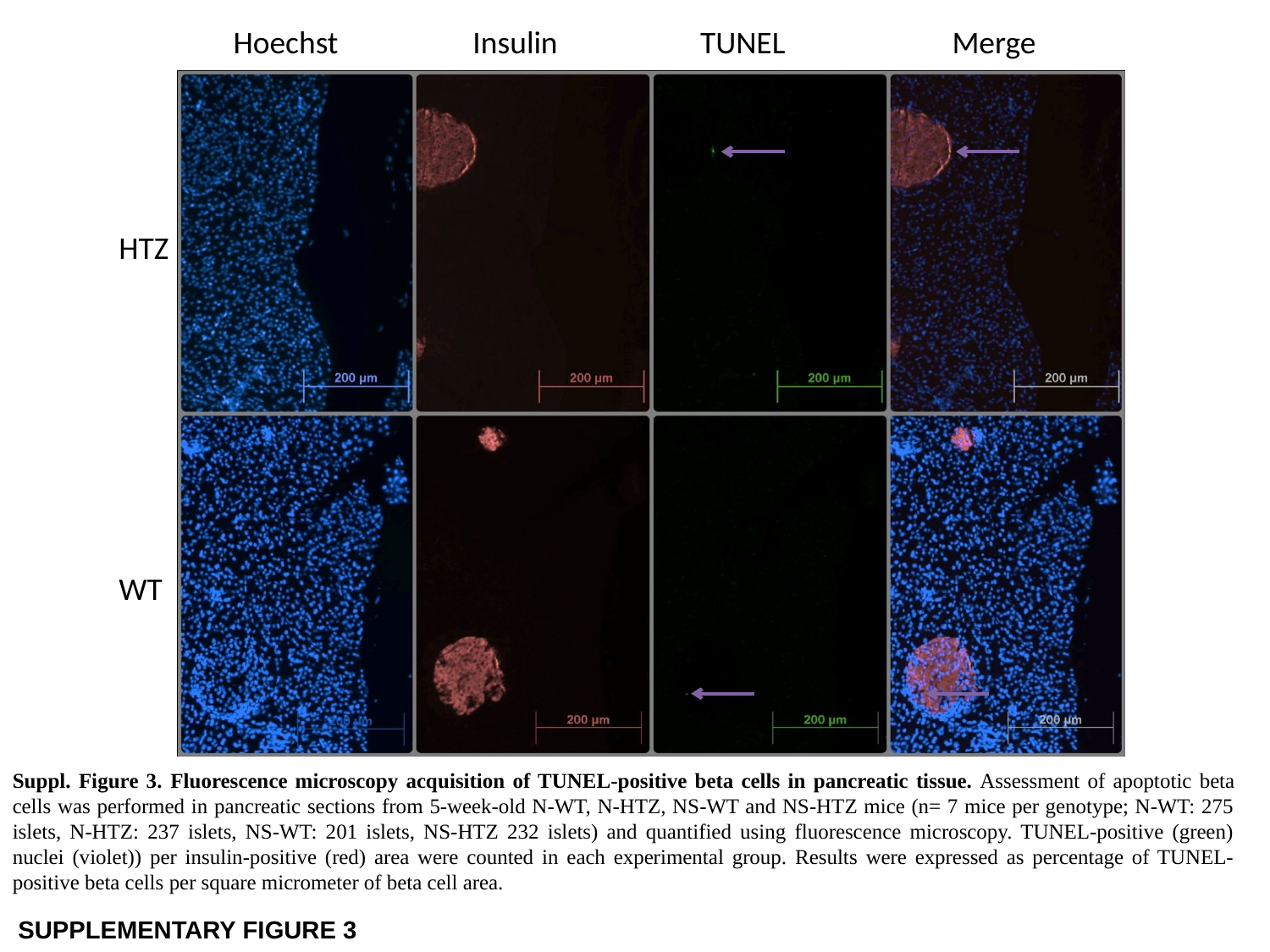

Hoechst	 Insulin	 TUNEL		Merge
HTZ
WT
Suppl. Figure 3. Fluorescence microscopy acquisition of TUNEL-positive beta cells in pancreatic tissue. Assessment of apoptotic beta cells was performed in pancreatic sections from 5-week-old N-WT, N-HTZ, NS-WT and NS-HTZ mice (n= 7 mice per genotype; N-WT: 275 islets, N-HTZ: 237 islets, NS-WT: 201 islets, NS-HTZ 232 islets) and quantified using fluorescence microscopy. TUNEL-positive (green) nuclei (violet)) per insulin-positive (red) area were counted in each experimental group. Results were expressed as percentage of TUNEL-positive beta cells per square micrometer of beta cell area.
SUPPLEMENTARY FIGURE 3
