# Supplementary material for: CDK11 Promotes Cytokine-Induced Apoptosis in Pancreatic Beta Cells Independently of Glucose Concentration and Is Regulated by Inflammation in the NOD Mouse Model
Source: Front Immunol. 2021 Feb 10;12:634797. doi: 10.3389/fimmu.2021.634797 (PMC7923961; doi:10.3389/fimmu.2021.634797)
Supplement: Supplementary file 3 [file Table_1.pdf]

Supplementary Table 1. Previous cdk11 mRNA microarray studies.

	**Genebank**	**NOD/SCID : NOD mRNA ratio**
Experiment ID	Accession #	Normalized values
Experiment 1	NM_007661.3	2,77
Experiment 2	NM_007661.3	2,05
average	Cdk11	2,4

Supplementary Table 1. mRNA microarray studies. Two populations of mRNAs from pancreatic islet endocrine cells (PECs) were compared as follows: the first population was obtained from non-diabetic insulitis-affected 11-week-old NOD female mice; therefore, these mice were at the edge of developing T1D; and the second population was obtained from non-diabetic insulitis-free 11-week-old NOD/SCID female mice. Single cell suspensions of pancreatic islets from 11-week-old NOD and NOD/SCID female mice were obtained, stained for CD45-PE (hematopoietic marker) and 7AAD (7 amino-actinomycin D, a marker of dead cells), and submitted to fluorescence activated cell sorting (FACS) (Becton Dickinson) to sort CD45-7AAD- (live islet endocrine cells). mRNA was isolated from CD45-7AAD- cells from both types of mice, i.e., NOD and NOD/SCID, using the TRIzol method (Invitrogen, Carlsbad, CA, USA). Subsequently, cDNA synthesis and subtractive hybridization were performed using the PCR-Select cDNA Subtractive Kit (Clontech, California, USA). The subtractive hybridization was performed to obtain cDNAs that are specifically expressed in each of the experimental cDNA samples, i.e., NOD and NOD/SCID. The NOD-enriched and NOD/SCID-enriched cDNA probes were used to hybridize to the microarray slides. cDNA mouse microarray slides (4.6K cDNA: 4608 mouse cDNAs spotted on a glass slide in duplicate) were provided by the Keck Microarray Facility at Yale University (New Haven, CT, USA). The full list of genes included in this microarray is available upon request to the Keck Microarray Facility at Yale University. The hybridization of both NOD- and NOD/SCID-enriched fluorescently labeled probes to the glass slides was performed twice; the microarray slide scanning, raw data collection, background-subtracted data analysis, and statistical analysis were performed by the Keck Microarray Facility at Yale University (New Haven, CT, USA). Only those cDNAs that showed an expression shift of two-fold or higher were considered differentially expressed. A summary of the normalized data relative to cdk11 from both hybridization experiments is included in the table above.
