# Supplementary material for: CDK11 Promotes Cytokine-Induced Apoptosis in Pancreatic Beta Cells Independently of Glucose Concentration and Is Regulated by Inflammation in the NOD Mouse Model
Source: Front Immunol. 2021 Feb 10;12:634797. doi: 10.3389/fimmu.2021.634797 (PMC7923961; doi:10.3389/fimmu.2021.634797)
Supplement: Supplementary file 4 [file Table_2.pdf]

Supplementary Table 2. Cumulative incidence of diabetes in 30 week old NODSCID mice hemideficient in CDK11

Genotype	% Cumulative incidence of diabetes by 30 weeks of age	Total number of mice observed
WT	0	n=16
HTZ	0	n=15
